# Proarrhythmic effect of radiofrequency catheter ablation on the right ventricular moderator band

**DOI:** 10.1002/joa3.12752

**Published:** 2022-07-09

**Authors:** Roy M. John, Jonathan Willner

**Affiliations:** ^1^ Stanford School of Medicine Stanford University Palo Alto California USA; ^2^ Department of Cardiology Northshore University Hospital Manhasset New York USA

**Keywords:** moderator band, premature ventricular contractions, pro arrhythmia, RF ablation, ventricular fibrillation

## Abstract

Following ablation on the RV moderator band for suppression of monomorphic PVCs, recurrent VT and VF were triggered by the same PVC at shorter coupling intervals. This is likely a pro arrhythmic effect of ablation on the moderator band.

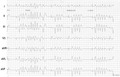

## 1 SPOTLIGHT

**FIGURE 1:**
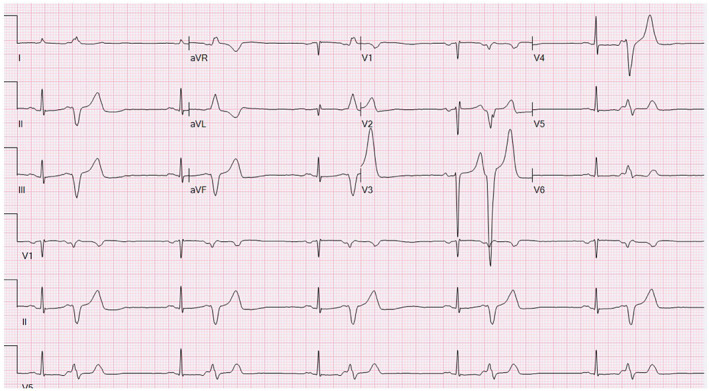
Baseline ECG showing PVCs at bigeminal frequency.

A 59‐year‐old man presented with fatigue due to bigeminal premature ventricular contractions (PVCs) (Figure 1). He had a history of hypertension and end‐stage diabetic nephropathy requiring chronic hemodialysis. An echocardiogram showed a global hypokinesis and LV ejection fraction (LVEF) of 40%. Cardiac magnetic resonance imaging showed no late gadolinium enhancement and angiography showed mild irregularity but no occlusive coronary artery disease. He was referred for electrophysiological study and ablation of PVCs.

**FIGURE 2:**
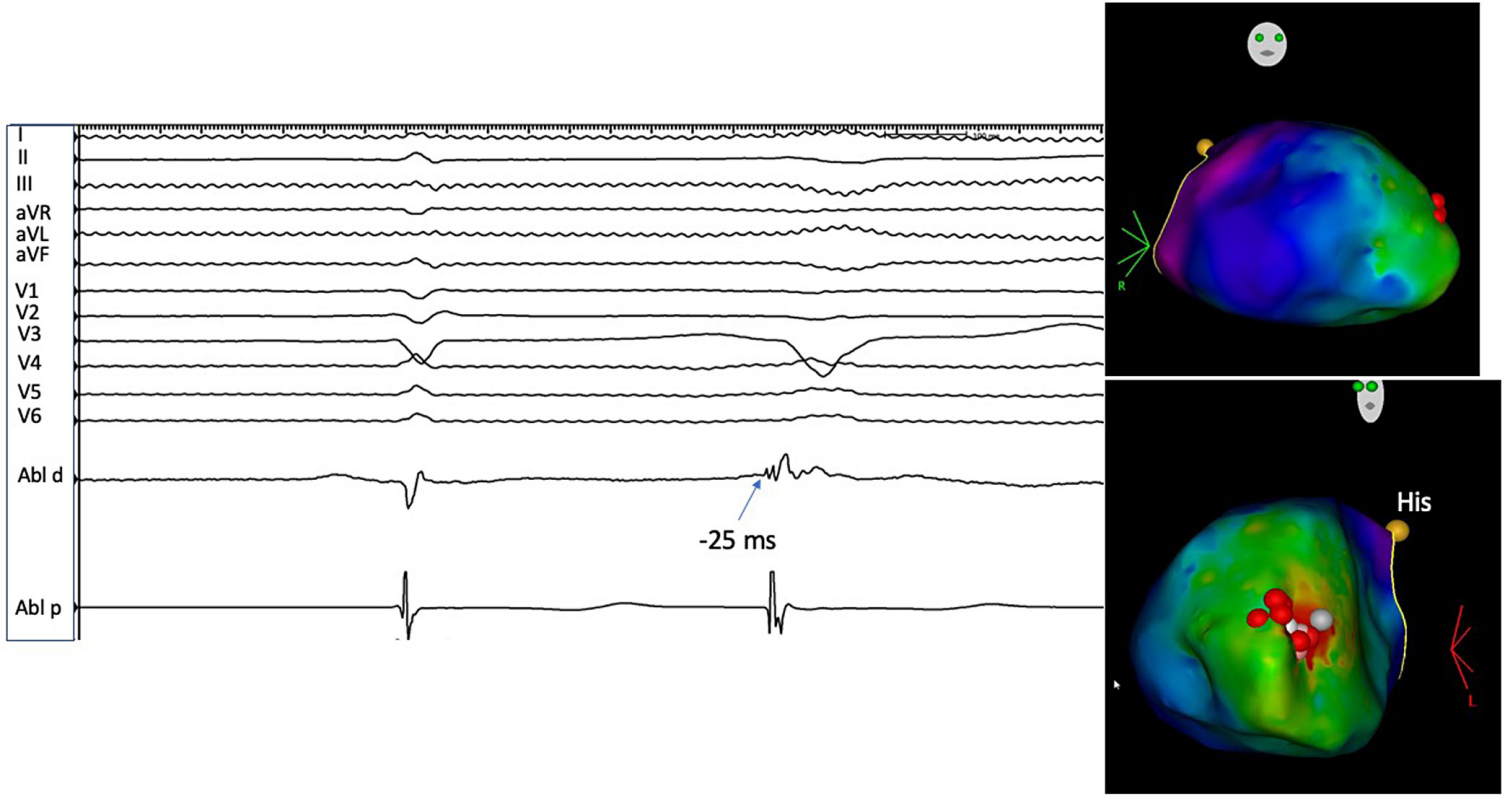
Electrogram at the site of initial ablation was 25 ms presystolic (left panel). Activation maps of the right ventricle (right panel) in the right anterior oblique (top image) and left anterior oblique (bottom image) show the earliest activation at the distal septum that corresponded to the septomarginal trabeculations initiating the moderator band. Red dots depict ablation lesions.

Bigeminal PVCs were present at baseline with a coupling interval of 480 ms. PVC morphology was left bundle branch block pattern (LBBB) with precordial QRS transition in V4 and left superior axis and QRS duration of 140 ms (Figure 1). Intracardiac echocardiography was used to create a three‐dimensional (3‐D) shell of the right ventricle (RV), septal and anterior papillary muscle and the trabeculations from the mid/apical septum to the anterior papillary muscle was traced as the moderator band. A clear band traversing the chamber was not visualized. Activation mapping of the PVCs showed the earliest activation at the trabeculation close to the septum with early activation that was 25 ms presystolic (Figure 2). A clear Purkinje potential could not be recorded. RF ablations (*n* = 6) using 30–40 W for a maximum of 30 s and contact force of 7–10 g at the site suppressed PVCs (Figure 2). The ablation also resulted in a right bundle branch block (RBBB). No further PVCs were observed over 30 min. Programmed ventricular stimulation with triple extra‐stimulation at 600 and 400 ms drive cycle lengths failed to induce ventricular arrhythmias. The patient returned to a telemetry ward.

**FIGURE 3:**
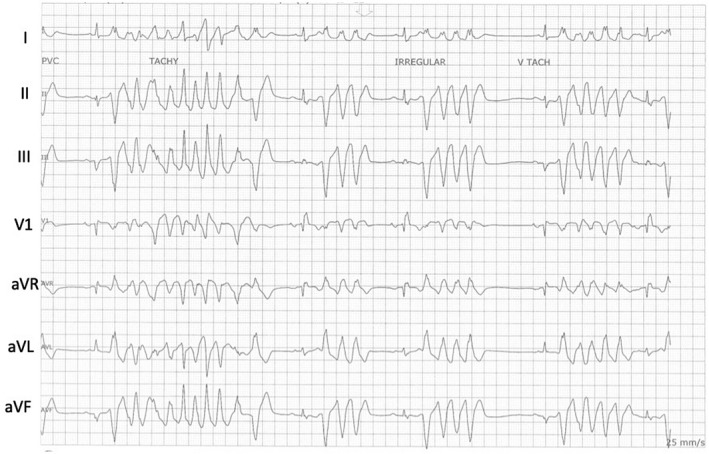
Runs of repetitive polymorphic VT triggered by the PVCs of the same morphology at the initial PVC (LBBB superior axis).

**FIGURE 4:**
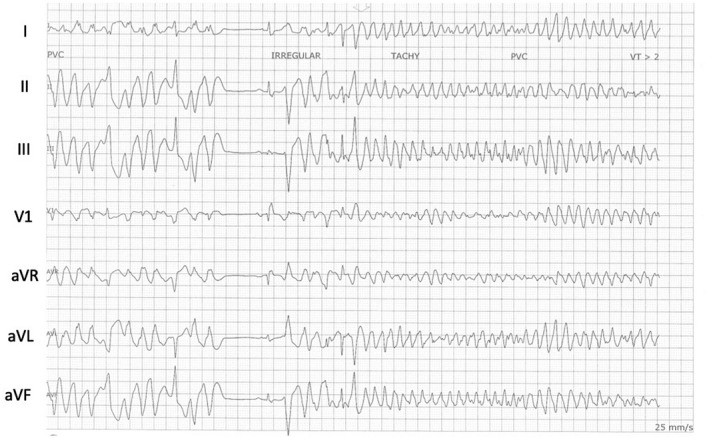
Ventricular fibrillation triggered by the same PVCs of the same morphology as the initial PVC.

Six hours after the ablation, PVCs of the same morphology returned with a shorter coupling interval of 240 ms and runs of non‐sustained ventricular tachycardia (VT) (Figure 3). Intravenous lidocaine was begun for suppression. Ventricular fibrillation (VF) was triggered by the same PVC (Figure 4) and required resuscitation with repeated external defibrillation and the use of intravenous procainamide. No alterations in serum electrolytes were recorded. There was no evidence for ST‐segment changes on ECG. Temporary transvenous pacing was implemented to overdrive pace and suppress PVC. The patient was taken back to the electrophysiology lab where intravenous antiarrhythmic drugs were discontinued. The pacing was discontinued. No PVCs were evident. Based on prior mapping data, the septal aspect of the moderator band was targeted for ablation using a 23 mm Arctic Front IV cryoballoon (Medtronic). A multipolar Achieve catheter (Medtronic) was advanced beyond the moderator band to the apex of the RV, and the cryoballoon was positioned at the septal end of the moderator band. Two lesions were applied for 180 and 140 s with the lowest temperature recordings of −55 and −45°C, respectively. The cryoballoon was repositioned more laterally toward the anterior papillary muscle and further two lesions were created. The repeat voltage map showed a small area of low voltage in the apical septal area without the involvement of the RV apex. Post ablation, a period of observation of 2 h and stimulation with epinephrine and programmed ventricular stimulation were performed. No arrhythmias were observed.

The patient had no further ventricular arrhythmia on telemetry for 48 h. He was implanted with a subcutaneous cardioverter defibrillator prior to hospital discharge after screening for T wave oversensing. During a follow up of 3 months, no further arrhythmias have been recorded. Repeat echocardiography has shown improved systolic function with LVEF of 55% and normal RV systolic function.

We report here, a case of radiofrequency (RF) ablation at a septal aspect of the RV moderator band for suppression of frequent monomorphic PVCs. After initial suppression following RF application, PVCs recurred at a shorter coupling interval, with frequent repetitive non‐sustained VT leading to sustained VF. Given his prior history of stable PVCs and non‐sustained VT, the occurrence of short coupled PVCs triggering VF has to be considered a pro‐arrhythmic effect of RF ablation on the structures that comprise the moderator band. The subsequent use of a cryoballoon to ablate over the course of the moderator band probably resulted in or contributed to the complete and durable suppression of ventricular arrhythmias.

The RV moderator band is increasingly being recognized as a source for PVCs and PVC‐mediated VF.^1^, ^2^, ^3^ The moderator band extends from the septomarginal trabeculations on the RV side of the septum and extends to the anterior papillary muscle. Its structure varies from being short and thick to a strand of muscle extending across the distal half of the RV cavity. It carries within it, fascicles of the right bundle that serve to rapidly activate the RV‐free wall. The moderator band is abundant in Purkinje tissue that is insulated from the myocardium until peripheral arborization to the RV‐free wall. Monomorphic PVCs, non‐sustained monomorphic VT and VF are documented arrhythmias originating from the moderator band.^1^, ^2^ The benign PVCs usually have a coupling interval in excess of 400 ms as in the initial arrhythmia of the patient in this case.^4^ When PVCs trigger VF, coupling intervals are typically short, less than 400 ms.^3^, ^4^

In the present case, the exact mechanism for the emergence of frequent short‐coupled PVCs after RF ablation for transient suppression of the more benign form of arrhythmia is speculative. The creation of a RBBB may have abolished an overdrive suppressive phenomenon. Alternatively, an injury response of the Purkinje tissue as seen in the early phase of acute myocardial infarction may have been responsible.^5^ Such aggravation of Purkinje fiber‐mediated arrhythmias following ablation has not been previously documented and should be a consideration when inadequate suppression is achieved and PVCs re‐emerge with coupling interval shortening.

Because of the varying anatomy of the moderator band, the structure may not be well visualized even with intracardiac echocardiography. Ablation with an RF catheter can be difficult due to the inability to maintain consistent contact. The use of a cryocatheter or a cryoballoon can achieve better stability.^2^ In the present case, the use of a 23 mm Arctic Front IV Cryoballoon enabled a more diffuse ablation all along the course of the MB. RV function remained unchanged by transthoracic echocardiography.

## CONFLICT OF INTEREST

Roy M. John, MD: Lecture Honorarium, Abbott Inc.
